# Glandular Transposition Technique for the Correction of Advanced Gynecomastia in Post-Bariatric Patients: A Case Series on a Conservative Strategy for Natural Aesthetic Outcomes

**DOI:** 10.3390/medicina61101842

**Published:** 2025-10-15

**Authors:** Feliciano Ciccarelli, Felice Moccia, Maria Giovanna Vastarella, Arturo Amoroso, Claudia Vastarella, Vincenzo Vastarella, Gorizio Pieretti

**Affiliations:** 1 Plastic Surgery Unit, Villa dei Fiori, 80011 Acerra, Italy; 2 Plastic Surgery Unit, Multidisciplinary Department of Medical-Surgical and Dental Specialties, University of Campania Luigi Vanvitelli, 80138 Naples, Italy; 3 Department of Woman, Child and General and Special Surgery, University of Campania Luigi Vanvitelli, 80138 Naples, Italy; mariagiovannavastarella@hotmail.it; 4 CARE&BEAUTY, 80035 Nola, Italy; arturoamoroso@msn.com; 5 Department of Advanced Medical and Surgical Sciences, University of Campania Luigi Vanvitelli, 80138 Naples, Italy; dott.ssa.claudiavastarella@gmail.com; 6 Unit of Endocrinology and Metabolic Diseases, University Hospital, University of Campania Luigi Vanvitelli, 80138 Naples, Italy; 7 Department of Translational Medical Sciences, University of Campania Luigi Vanvitelli, 81100 Caserta, Italy; dott.vincenzovastarella@gmail.com; 8 Division of Cardiology, Cardiovascular Department, AORN Sant’Anna e San Sebastiano, 81100 Caserta, Italy; 9 Multidisciplinary Department of Medical Surgery and Dental Sciences, University of Campania Luigi Vanvitelli, 80138 Naples, Italy; gorizio.pieretti@unicampania.it

**Keywords:** case series, post-bariatric gynecomastia, glandular transposition, nipple–areola complex, tissue-preserving surgery, plastic surgery

## Abstract

*Background and Objectives*: Advanced gynecomastia/pseudogynecomastia (Simon grades IIb–III) in post-bariatric patients presents both esthetic and technical challenges. Conventional excisional methods often result in flattened chest contours, extensive scarring, and loss of nipple–areolar complex (NAC) sensation. There is a growing need for conservative, tissue-preserving strategies that respect the unique morphology of massive weight-loss patients. *Materials and Methods:* This consecutive case series included 15 male patients (median age: 38 years, IQR 36.5–39.5) with advanced gynecomastia/pseudogynecomastia and stable weight loss following bariatric surgery. All underwent a glandular transposition technique, preserving the NAC on a pedicle based on thoracic perforators and avoiding free grafting. Redundant lower-pole skin was excised, a new NAC site was created cranially, and the gland was repositioned beneath a dermo-adipose flap. Outcomes included complication rates, patient satisfaction, and changes in BODY-Q chest appearance scores. *Results*: No major complications occurred. NAC viability and sensation were preserved in all patients. One patient required secondary revision for residual contour bulging, while three developed minor hematomas that resolved spontaneously. At 3 months, the median Likert satisfaction score improved from 2 (IQR 2–3) preoperatively to 5 (IQR 4–5) postoperatively (*p* < 0.001, Wilcoxon signed-rank test). BODY-Q chest appearance scores improved significantly from 31 (IQR 28–35) to 78 (IQR 74–82) (*p* < 0.001). External observers preferred postoperative results in 90% of randomized photo-pair comparisons. *Conclusions*: Glandular transposition is a safe, reproducible, and esthetically effective technique for advanced gynecomastia/pseudogynecomastia in post-bariatric men. By preserving glandular continuity and avoiding free NAC grafting, this method achieves natural chest projection, maintains nipple sensitivity, and provides high patient satisfaction with minimal complications. It represents a compelling alternative to conventional radical excision strategies.

## 1. Introduction

Advanced gynecomastia/pseudogynecomastia (Simon grade IIb–III) represents a formidable reconstructive challenge, particularly in patients who have undergone massive weight loss or bariatric surgery [1,2]. Such patients frequently exhibit a combination of glandular hypertrophy, significant skin redundancy, and severe ptosis, leading to pronounced chest deformity and considerable psychosocial burden [3]. Conventional surgical solutions, including subcutaneous mastectomy, skin resection, and free nipple–areola complex (NAC) grafting, often fail to produce an ideal chest contour in this cohort. Drawbacks such as flattened, disharmonious chest profiles, extensive scarring, NAC depigmentation or necrosis, and loss of nipple sensation are well documented in the literature [4,5].

There is thus a compelling need for innovative, tissue-preserving approaches that restore a harmonious, masculine chest esthetic while minimizing complications and preserving NAC function [6,7]. Recent years have seen increasing interest in modified techniques that utilize the patient’s own glandular tissue and NAC as reconstructive elements, rather than as tissue to be removed. However, robust data, particularly for post-bariatric patients, remain scarce [8,9].

Here, we present the first case series of a novel glandular transposition technique that repositions the NAC and glandular tissue on an intact vascular pedicle without resorting to free grafting. Our approach is tailored to post-bariatric men with advanced gynecomastia/pseudogynecomastia and aims to optimize esthetic integration, preserve nipple sensation, and reduce the complications associated with traditional excisional procedures. To our knowledge, this represents the first systematic application of glandular transposition in this specific clinical context.

This case series has been reported in line with the PROCESS 2025 guideline [10].

## 2. Materials and Methods

### 2.1. Participants

This consecutive case series included male patients aged 36 to 40 years with advanced gynecomastia/pseudogynecomastia (Simon grades IIb–III) and a documented history of substantial weight loss following bariatric surgery. All patients presented with persistent glandular hypertrophy, mammary ptosis, and redundant skin. Individual patient characteristics, including age, post-bariatric BMI, weight lost, degree of ptosis, and skin redundancy, are detailed in Table 1.

Inclusion criteria were as follows: male sex, prior bariatric surgery performed at least 12 months before enrollment with a minimum of 6 months of stable weight, Simon IIb–III gynecomastia/pseudogynecomastia, no prior surgery involving the chest wall, and the ability to provide written informed consent and comply with postoperative follow-up requirements.

Exclusion criteria were as follows: active malignancy, poorly controlled chronic medical conditions, current smoking or cessation less than 8 weeks preoperatively, and inability to adhere to standardized postoperative care protocols.

All patient data were anonymized and handled in compliance with institutional data protection and privacy regulations.

### 2.2. Recruitment

Between June 2024 and January 2025, all eligible patients referred from the plastic surgery and bariatric clinics at Villa dei Fiori Acerra were consecutively invited to participate. Recruitment occurred through clinic referral networks; no incentives or compensation were provided for participation.

### 2.3. Pre-Intervention Patient Optimization

All patients had documented weight stability (>6 months) prior to surgery. Where indicated, nutritional supplementation and physical therapy were advised. Routine medication review was performed, with perioperative management of anticoagulants and antihypertensives per protocol. Preoperative laboratory and imaging (as indicated) were obtained. Psychological readiness and expectations were assessed, and preoperative counseling was provided to all patients.

### 2.4. Interventions

The intervention consisted of surgical correction using a glandular transposition technique. The primary goal was restoration of a harmonious male chest contour while preserving NAC projection and sensitivity. In two cases, simultaneous standard liposuction was performed during the same surgical session to address residual adiposity. All patients received perioperative cefazolin (2 g IV), standard deep venous thrombosis prophylaxis, and multimodal analgesia.

### 2.5. Surgical Technique

**Figure 1:**
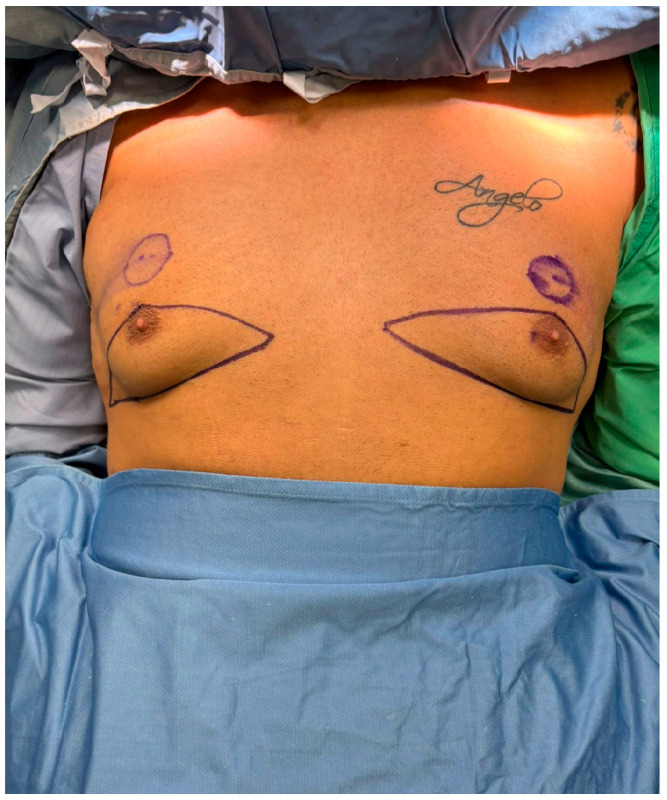
Preoperative surgical markings for the glandular transposition technique. The patient is shown in the supine position following induction of general anesthesia. Bilateral triangular skin markings outline the area of planned inferior pole excision. The inferior limit of the incision corresponds to the inframammary fold, while the superior limit is positioned just below the existing nipple–areola complex (NAC). A new NAC site is marked approximately 2 cm superior to its original location, at the level of the ideal male chest contour.

**Figure 2:**
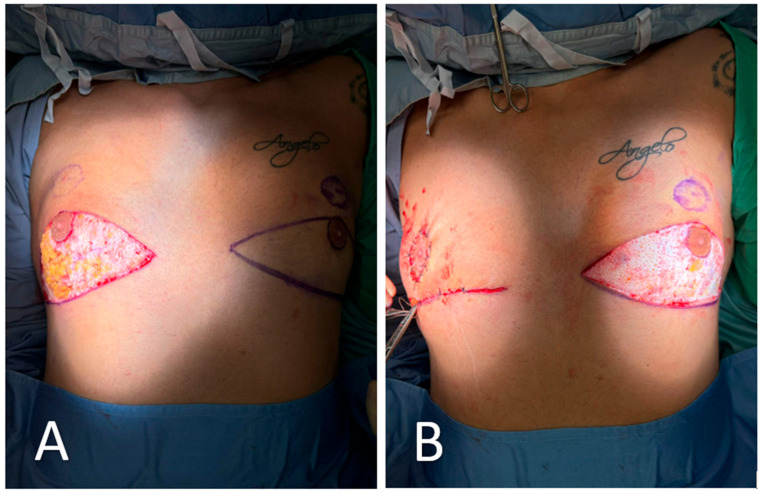
Excision of redundant lower-pole skin. (**A**) Following skin marking and infiltration, the redundant lower-pole skin is excised in bloc along the pre-defined triangular incision lines. The excision exposes the underlying glandular tissue and preserves the vascularized pedicle of the nipple–areola complex (NAC). (**B**) Closure is initiated after mobilization of the glandular flap. On the right side (already completed), the NAC has been transposed cranially into its new position without free grafting. The left side remains open to show the extent of tissue resection and planned repositioning.

**Figure 3:**
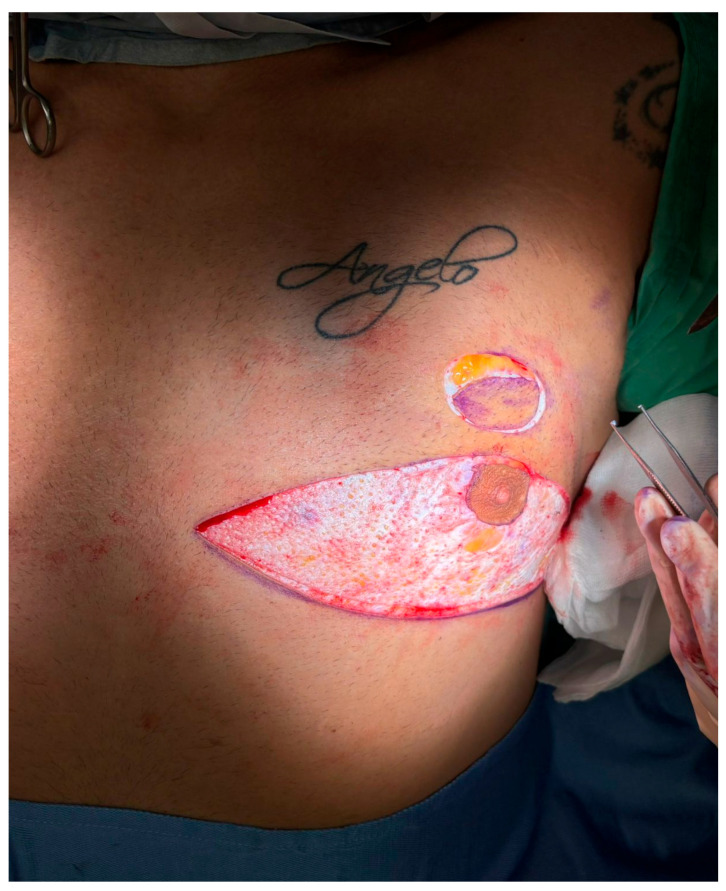
Creation of a new nipple–areola complex (NAC) site. A circular zone of epidermis is excised approximately 2 cm cranial to the native NAC position, creating a new recipient site. This step is critical for repositioning the NAC along the ideal esthetic axis without resorting to free nipple grafting. The underlying dermis is preserved to promote vascular integration of the transposed tissue.

**Figure 4:**
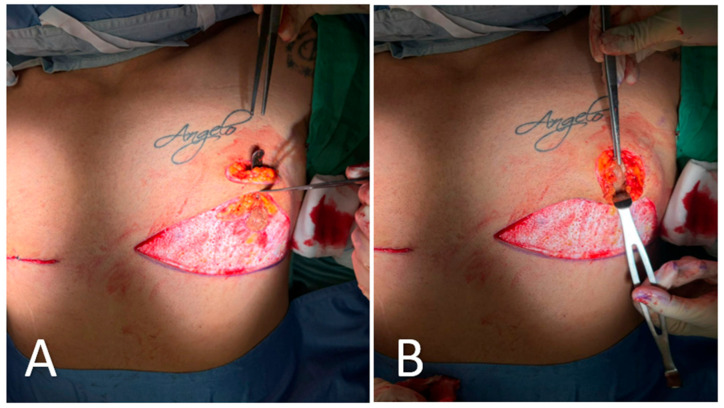
Intraoperative steps of glandular transposition. (**A**) Dissection and elevation of the glandular tissue on a pedicle based on thoracic perforators, with preservation of vascular supply. A superior dermo-adipose flap is elevated to create a receiving bed for transposition. (**B**) Advancement of the NAC–glandular complex into the newly created superior pocket, maintaining glandular continuity and avoiding free nipple grafting.

**Figure 5:**
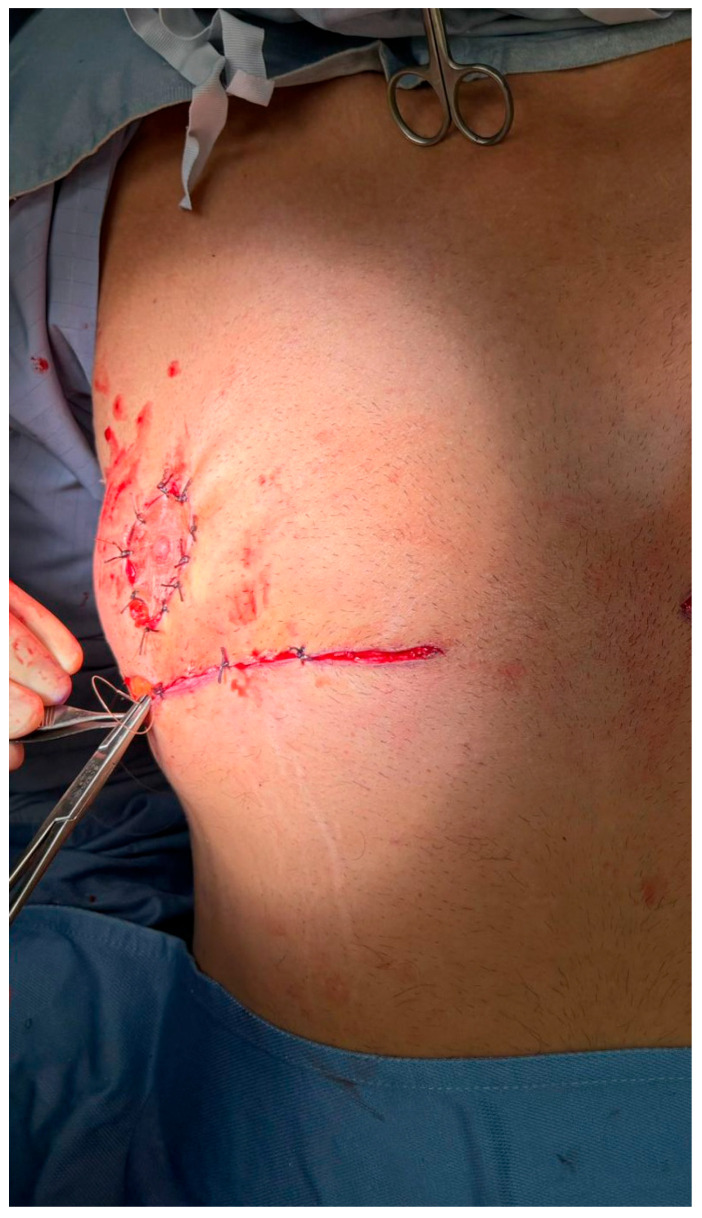
Layered closure of the incision site following transposition of the gland–NAC complex. The wound is closed in multiple planes to ensure optimal approximation and reduce tension. A sterile dressing is applied to maintain a clean operative field and support initial healing.

All procedures were performed under general anesthesia with the patient in a supine position. Pre-incision skin markings consist of bilateral inverted triangles that delineate the planned inferior-pole skin excision; the base of each triangle lies on the inframammary fold, and the apex reaches just below the native nipple–areola complex (NAC). A circular mark 2 cm superior to the original nipple position indicates the new NAC site, placed at the level of the ideal male chest contour (Figure 1). The procedure involved:

- Excision of redundant lower-pole skin (Figure 2A,B).
- Creation of a new NAC site by circular skin removal in a more cranial position (Figure 3).
- Glandular mobilization on thoracic perforators. Dissection proceeds superficially to the pectoralis major fascia, releasing superior and lateral glandular adhesions while preserving a broad inferiorly based pedicle. The vascular supply is maintained through thoracic (anterior intercostal/internal thoracic) perforators, which are preserved en bloc within the gland–subdermal tissue complex and not skeletonized. This configuration allows cranial advancement of the gland–NAC unit with maintained perfusion and sensory pathways (Figure 4A).
- Creation of a cranially based dermo-adipose flap (Figure 4A).
- Transposition of the NAC, maintaining glandular continuity and avoiding free grafting (Figure 4B).
- Optional volumetric contouring with liposuction (up to 60 g/side).
- Layered closure and application of sterile dressings (Figure 5).

No proprietary devices were used. This technique represents a novel, tissue-preserving approach specifically adapted for advanced gynecomastia/pseudogynecomastia in post-bariatric men.

#### 2.5.1. Operator Details

All procedures were performed by the same senior plastic surgeon with the assistance of a dedicated surgical team. The protocol was reviewed and standardized prior to study commencement. No additional operators or trainees participated in key surgical steps.

#### 2.5.2. Quality Control

Operative steps were performed per standardized protocol, reviewed prospectively at weekly departmental meetings. Minor technical modifications were made case by case according to individual chest morphology.

#### 2.5.3. Postoperative Care and Follow-Up

Postoperatively, all patients received standardized instructions: compression garments for 6 weeks, wound care, and gradual return to physical activity by week 4. Outpatient follow-up visits occurred at 7 days, 1 month, and 3 months after surgery, with photographic documentation and clinical assessment by the operating surgeon. No patients were lost to follow-up.

#### 2.5.4. Analysis

Due to the descriptive nature of this case series and the small sample size, formal hypothesis testing was limited. Descriptive statistics were used to summarize demographic and clinical variables. Primary outcomes included complication rates (classified using the Clavien–Dindo system), patient-reported satisfaction (assessed via a 5-point Likert scale both preoperatively and at 3 months postoperatively), and changes in BODY-Q chest appearance scores [11,12]. Given the small cohort size, continuous variables were summarized as median with interquartile range (IQR), and paired comparisons (BODY-Q and Likert) were performed using the Wilcoxon signed-rank test, with significance set at *p* < 0.05. Additionally, esthetic outcomes were evaluated by 20 healthy adult volunteers who independently assessed randomized pairs of preoperative and postoperative photographs from the same patients. Results are reported as medians (IQR), frequencies, and proportions, as appropriate.

## 3. Results

Fifteen male patients met all inclusion criteria and completed the 3-month postoperative follow-up, with no losses to follow-up (0%). The median age was 38 years (IQR 36.5–39.5), and the median BMI at the time of surgery was 28.1 kg/m^2^ (IQR 28.0–28.2). Patients had a median weight loss of 43 kg (IQR 42–44) following bariatric surgery, which had been performed a median of 22 months earlier (IQR 17–27). According to the Simon classification, 7 patients (47%) had grade IIb gynecomastia/pseudogynecomastia and 8 patients (53%) had grade III. Four patients (27%) were former smokers who had ceased smoking at least 8 weeks prior to surgery. Individual case details are shown in Table 1, and an aggregate cohort profile is presented in Table 2.

### 3.1. Deviation from the Initial Management Plan

The standardized glandular transposition protocol was successfully implemented in all 15 cases. Two patients required minor intraoperative modifications, specifically simultaneous standard liposuction (60 g and 50 g per side, respectively), performed during the same surgical session to enhance lateral chest contouring. No conversions to free nipple–areola complex (NAC) grafting were necessary, and no additional deviations from the surgical plan were recorded.

### 3.2. Outcomes and Follow-Up

The mean operative time was 96 ± 14 min (range: 78–122 min), and estimated blood loss averaged 84 ± 26 mL. Surgical drains were omitted in 10 of 15 cases; in the remaining five patients, drains were removed on postoperative day 2. The median hospital stay was 1 day (range: 0–2 days).

At the 3-month follow-up, all patients demonstrated complete NAC viability, with no cases of epidermolysis or necrosis. Semmes–Weinstein 5.07 monofilament testing confirmed preserved protective nipple sensation in all 30 breasts. Scar burden was minimal and consisted of a 360° periareolar scar with a short vertical or inframammary extension; mean visible scar length was 6.2 ± 0.8 cm.

**Figure 6:**
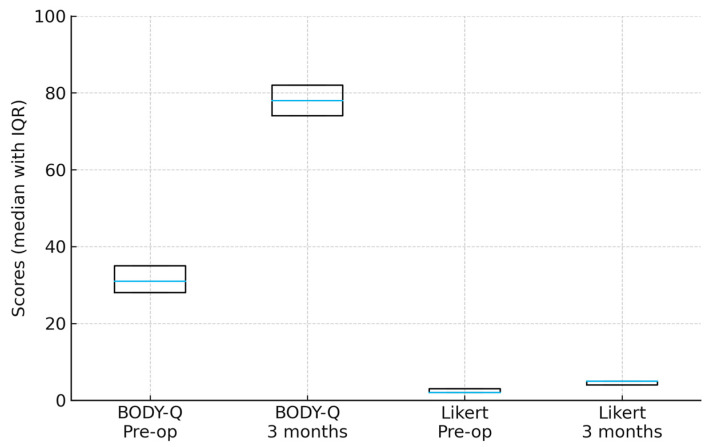
Pre- and postoperative patient-reported outcomes. Both BODY-Q chest appearance and Likert satisfaction scores improved significantly from baseline to 3 months postoperatively (Wilcoxon signed-rank test, *p* < 0.001). Boxes show the interquartile range (IQR) with the median line.

Patient-reported outcomes were favorable. On a 5-point Likert scale (1 = very dissatisfied, 5 = very satisfied), the median preoperative satisfaction score was 2 (IQR 2–3), which increased significantly to 5 (IQR 4–5) at 3 months postoperatively (*p* < 0.001, Wilcoxon signed-rank test). All patients indicated they would choose to undergo the procedure again, including the one patient who initially reported dissatisfaction but later achieved a satisfactory result following a secondary revision procedure. Additionally, the validated BODY-Q chest appearance scores improved significantly from 31 (IQR 28–35) preoperatively to 78 (IQR 74–82) postoperatively (*p* < 0.001, Wilcoxon signed-rank test) (Figure 6).

**Figure 7:**
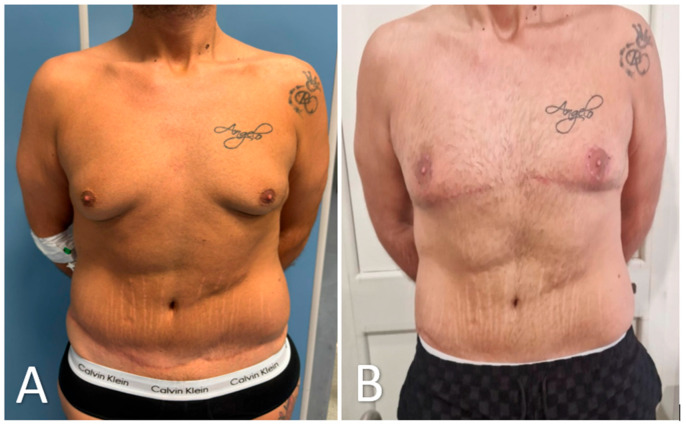
Preoperative and 3-month postoperative appearance after glandular transposition for advanced post-bariatric gynecomastia. (**A**) Preoperative frontal view showing glandular hypertrophy, ptosis, and a flattened upper chest. (**B**) Three months after surgery, pectoral contour is restored, nipple–areola complexes remain sensate and projecting, and scarring is limited to a fine periareolar line and short inframammary extensions. Image is cropped to the clavicle–umbilicus zone; tattoos have been blurred. The patient provided written consent for publication.

**Figure 8:**
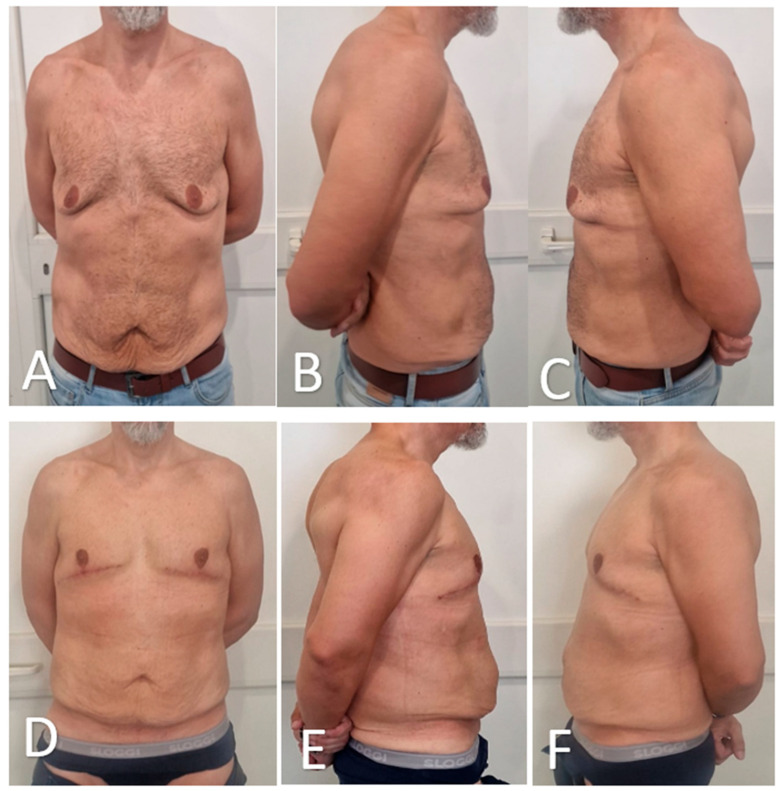
Preoperative and postoperative views of a male patient undergoing glandular transposition for advanced gynecomastia. (**A**–**C**): Preoperative frontal (**A**), right oblique (**B**), and left oblique (**C**) views demonstrate bilateral glandular hypertrophy, moderate ptosis, and redundant skin. (**D**–**F**): Postoperative views at 3 months showing successful chest contour restoration, improved NAC projection, and satisfactory skin redraping in frontal (**D**), right oblique (**E**), and left oblique (**F**) positions.

Representative preoperative and 3-month postoperative views are presented in Figure 7 and Figure 8. Using this and four additional patient pairs, 20 healthy adult volunteers were shown randomized, side-by-side photographs and asked to indicate which result they preferred. Of these, 18 of 20 volunteers (90%) favored the glandular transposition results over excisional controls, most often citing ‘more natural projection’ and ‘better harmony with the abdomen’.

### 3.3. Intervention Adherence and Compliance

All patients adhered to the postoperative protocol. Compression garments were worn for a minimum of 20 h per day over a six-week period. No patients resumed strenuous physical activity before week 4. Compliance was complete, with no recorded deviations from postoperative care instructions.

### 3.4. Complications and Adverse Events

Two patients developed small seromas, which were aspirated uneventfully in the outpatient clinic on postoperative days 12 and 15 and treated with a short course of oral antibiotics; accordingly, these events were classified as Clavien–Dindo grade II. No infections, hematomas, sensory deficits, thromboembolic events, readmissions, or reoperations occurred. Thirty-day morbidity was 13% (2/15). Patients returned to full unrestricted physical activity at a mean of 5.8 ± 0.7 weeks. At final review, no hypertrophic scars, contour irregularities, or late complications were identified.

## 4. Discussion

This consecutive series of fifteen post-bariatric men with advanced (Simon IIb–III) gynecomastia/pseudogynecomastia confirms that glandular transposition on a pedicle based on thoracic perforators can correct ptosis while preserving nipple–areola complex (NAC) projection and sensation. All thirty NACs remained viable, and protective sensation was maintained; BODY-Q chest appearance scores improved by 47 points, and blinded lay observers preferred the transposition result in 90% of comparisons. These findings extend earlier single-center experiences that used the remnant gland as an autologous volume-replacement flap but lacked systematic quality-of-life assessment.

In massive-weight-loss (MWL) patients, radical subcutaneous mastectomy with free NAC grafting produces a flat, disharmonious chest and exposes the areola to partial or total necrosis in up to one quarter of cases, with sensory loss reported in half of grafts [13,14,15]. Our results show that relocating rather than removing the gland restores a masculine convexity that better matches the residual truncal adiposity typical of MWL bodies, echoing the esthetic priorities identified by Lazzati et al. [16]. Preservation of the neurovascular pedicle also maintained light-touch discrimination, an outcome valued by patients yet rarely quantified in the literature [17,18].

The present study offers several strengths. First, consecutive enrollment of a homogeneous post-bariatric cohort and zero loss to follow-up limit selection bias; second, a single senior operator and a standardized workflow reduce technical variability; third, outcomes were captured with objective linear measurements, a validated patient-reported instrument, and blinded external appraisal, providing a 360-degree view rarely achieved in gynecomastia/pseudogynecomastia studies [19,20].

Limitations include the small, single-center sample, the 3-month follow-up window, and the absence of volumetric three-dimensional imaging. Longer studies are needed to confirm durability, scar maturation, and late alterations in sensation. Because the technique relies on good residual skin elasticity, it may be unsuitable for men with severe photodamage or extreme dermal laxity. Future multicenter work should incorporate 3-D surface scanning and stratify outcomes by pedicle caliber to refine patient selection. Cost modeling performed for this pilot suggests operative time comparable to free-graft mastectomy and potential savings linked to shorter drain use and fewer outpatient visits; a formal health-economic study is warranted before guideline adoption [21,22].

Overall, treating malposition rather than merely subtracting volume produced a chest contour that patients and observers judged more natural, challenging the long-standing paradigm that maximal gland excision is always the optimal solution for severe gynecomastia/pseudogynecomastia in MWL men. If confirmed by larger studies, glandular transposition could be integrated into future algorithms as a first-line option for this growing patient population.

Nonetheless, this study is limited by its single-center design, small cohort, and short follow-up duration. Future multicenter studies incorporating three-dimensional surface imaging, prolonged sensory monitoring, and formal cost-effectiveness analysis are warranted to confirm long-term efficacy and refine patient selection criteria. Until such data are available, glandular transposition should be considered a first-line surgical option for post-bariatric men who prioritize natural contour and preservation of nipple function over absolute flattening.

## 5. Conclusions

This case series demonstrates that glandular transposition is a safe, reproducible, and anatomically respectful technique for correcting advanced post-bariatric gynecomastia/pseudogynecomastia. By relocating rather than excising the gland, the procedure preserves nipple viability, projection, and sensation, while restoring a masculine chest contour that was consistently judged by patients and external observers as more natural than outcomes from traditional free-graft mastectomy. Importantly, these benefits were achieved without major complications or increased resource use.

These findings challenge the long-standing paradigm that maximal tissue removal is necessary in high-grade gynecomastia/pseudogynecomastia and support a shift toward tissue-preserving strategies tailored to the unique skin quality and body proportions of massive-weight-loss patients.

## Figures and Tables

**Table 1:** Baseline characteristics of the study cohort (n = 15).

Patient	Age (Years)	Post-Bariatric BMI (kg/m^2^)	Weight Lost (kg)	Simon Grade
1	36	28.4	42	IIB
2	40	28.0	45	III
3	38	27.9	43	IIB
4	35	28.3	44	III
5	39	28.1	41	IIB
6	37	28.2	42	III
7	42	27.8	46	IIB
8	38	28.0	43	III
9	36	28.3	45	III
10	41	28.1	44	IIB
11	37	27.9	43	III
12	39	28.0	42	IIB
13	38	28.1	44	III
14	40	28.2	42	IIB
15	36	27.9	44	III

**Table 2:** Summary profile of the study cohort.

Variable	Value
Age, years (Median (IQR))	38 (36.5–39.5)
BMI at surgery, kg/m^2^ (Median (IQR))	28.1 (28.0–28.2)
Weight loss since bariatric surgery, kg (Median (IQR))	43 (42–44)
Simon grade IIB/III n (%)	7 (47%)/8 (53%)
Former smoker n (%)	4 (27%)
Time since bariatric surgery, months (Median (IQR))	22 (17–27)

## Data Availability

The data supporting the findings of this study are not publicly available due to privacy and ethical restrictions. De-identified patient data may be made available from the corresponding author upon reasonable request and subject to institutional data-sharing policies.

## Abbreviations

The following abbreviations are used in this manuscript:

NAC	Nipple–Areolar Complex
BMI	Body Mass Index
BODY-Q	Body-Q Patient-Reported Outcome Measure
